# Natal foraging philopatry in eastern Pacific hawksbill turtles

**DOI:** 10.1098/rsos.170153

**Published:** 2017-08-23

**Authors:** Alexander R. Gaos, Rebecca L. Lewison, Michael P. Jensen, Michael J. Liles, Ana Henriquez, Sofia Chavarria, Carlos Mario Pacheco, Melissa Valle, David Melero, Velkiss Gadea, Eduardo Altamirano, Perla Torres, Felipe Vallejo, Cristina Miranda, Carolina LeMarie, Jesus Lucero, Karen Oceguera, Didiher Chácon, Luis Fonseca, Marino Abrego, Jeffrey A. Seminoff, Eric E. Flores, Israel Llamas, Rodrigo Donadi, Bernardo Peña, Juan Pablo Muñoz, Daniela Alarcòn Ruales, Jaime A. Chaves, Sarah Otterstrom, Alan Zavala, Catherine E. Hart, Rachel Brittain, Joanna Alfaro-Shigueto, Jeffrey Mangel, Ingrid L. Yañez, Peter H. Dutton

**Affiliations:** 1 Department of Biology, San Diego State University, San Diego, CA, USA; 2 Graduate Group in Ecology, University of California Davis, Davis, CA, USA; 3 Marine Mammal and Turtle Division, Ocean Associates Inc., under contract to the Southwest Fisheries Science Center, National Marine Fisheries Service, National Oceanic and Atmospheric Administration, La Jolla, CA, USA; 4 Department of Biology, University of Texas at El Paso, El Paso, TX, USA; 5 ProCosta, San Salvador, El Salvador; 6 Marine Turtles Department, Fauna & Flora International, Managua, Nicaragua; 7 Instituto de Ciencias del Mar y Limnología, Unidad Académica Mazatlán, Universidad Nacional de Mexico, Mazatlán, Mexico; 8 Equilibrio Azul, Quito, Ecuador; 9 Grupo Tortuguero de las Californias, A.C, La Paz, Mexico; 10 Latin American Sea Turtles, Tibás, Costa Rica; 11 Conservación de Recursos Costeros y Marinos, Ministerio del Ambiente de Panamá, Panama City, Panama; 12 Marine Mammal and Turtle Division, Southwest Fisheries Science Center, National Marine Fisheries Service, National Oceanic and Atmospheric Administration, La Jolla, CA, USA; 13 Sistema Nacional de Investigación, Panama City, Panama; 14 Instituto de Investigaciones Científicas y Servicios de Alta Tecnología, Panama City, Panama; 15 Campamento Tortuguero Mayto, A.C., Mayto, Mexico; 16 World Wildlife Fund of Panama, Panama City, Panama; 17 Marine Ecology Department, Universidad San Francisco de Quito/Galapagos Science Center, San Cristóbal, Galapagos Archipelago, Ecuador; 18 Paso Pacifico, Managua, Nicaragua; 19 Unidad Sinaloa, Centro Interdisciplinario de Investigación para el Desarrollo Integral Regional, Sinaloa, Mexico; 20 Instituto Politécnico Nacional, Sinaloa, Mexico; 21 Red Tortuguera, A.C, Guayabitos, Mexico; 22 Akazul, La Barrona, Guatemala; 23 Marine Turtle Research Group, School of Biosciences, University of Exeter, Penryn, UK; 24 Marine Biology Department, Universidad Cientifica del Sur, Lima, Peru; 25 ProDelphinus, Lima, Peru; 26 Eastern Pacific Hawksbill Initiative, San Diego, CA, USA

**Keywords:** natal homing, life history, hatchling dispersal, spatial ecology, conservation genetics, juvenile

## Abstract

The complex processes involved with animal migration have long been a subject of biological interest, and broad-scale movement patterns of many marine turtle populations still remain unresolved. While it is widely accepted that once marine turtles reach sexual maturity they home to natal areas for nesting or reproduction, the role of philopatry to natal areas during other life stages has received less scrutiny, despite widespread evidence across the taxa. Here we report on genetic research that indicates that juvenile hawksbill turtles (*Eretmochelys imbricata*) in the eastern Pacific Ocean use foraging grounds in the region of their natal beaches, a pattern we term natal foraging philopatry. Our findings confirm that traditional views of natal homing solely for reproduction are incomplete and that many marine turtle species exhibit philopatry to natal areas to forage. Our results have important implications for life-history research and conservation of marine turtles and may extend to other wide-ranging marine vertebrates that demonstrate natal philopatry.

## 1. Introduction

Understanding movement patterns is an essential component of successful wildlife management [1,2]. The movement and dispersal of highly migratory marine organisms such as marine turtles have been studied for decades (e.g. [3,4]) and some broad-scale patterns have become widely accepted. For instance, it is generally believed that most post-hatchling marine turtles spend a multi-year period in open-ocean gyres before recruiting to neritic foraging grounds, but deviations from this pattern are known to exist for some species and populations [5–9]. An abundance of research has also shown that once reaching sexual maturity, which can take between 10 and 50 years [9], most turtles return to the vicinity of their natal beaches to nest or reproduce (i.e. natal homing *sensu* [10]).

The specific dispersal behaviour of marine turtles to foraging grounds in relation to natal nesting beaches, termed rookeries, is not defined [11]. Particle simulation modelling and satellite tracking of post-hatchling turtles suggest a range of dispersal pathways over thousands of kilometres, indicating settlement in foraging areas can occur over extremely large spatial scales (e.g. [12–15]). Genetic research has demonstrated that foraging grounds are typically made up of individuals from multiple rookeries, further highlighting the potentially diffuse distribution of post-hatchling turtles [11]. Recent studies have found that juvenile loggerhead (*Caretta caretta*), green (*Chelonia mydas*) and hawksbill (*Eretmochelys imbricata*) turtles in the Atlantic actively home to foraging grounds in the vicinity of their natal habitats [16–22]. Despite evidence supporting this homing pattern across several marine turtle species, current literature on general marine turtle life-history has not fully recognized philopatry to natal areas during early life stages or explored potential conservation implications of this pattern [9,23].

In this study, we analysed mitochondrial DNA (mtDNA) sequences of juvenile and adult hawksbills from the eastern Pacific Ocean to evaluate the species' distribution at foraging grounds relative to natal rookeries and to assess the evidence of foraging philopatry to natal areas. Hawksbill turtles were considered virtually absent from the eastern Pacific Ocean as recently as 2007 [24] and remain as one of the world's most threatened marine turtle regional management units [25]. Although extensive field research over the past 10 years has revealed important hawksbill rookeries and foraging grounds, there are still considerable gaps in our understanding of dispersal of post-hatchling turtles to foraging areas and how rookeries contribute to foraging grounds. Additionally, several unique life-history characteristics have been discovered for hawksbills in this ocean region that could potentially influence the dispersal of hatchlings and rookery (i.e. nesting colony) contributions, including highly neritic habitat use, restricted home ranges and limited migrations [26–28], heightening interest in genetic research of hawksbills at foraging grounds.

## 2. Material and methods

### 2.1. Sample collection and processing

**Figure 1.**
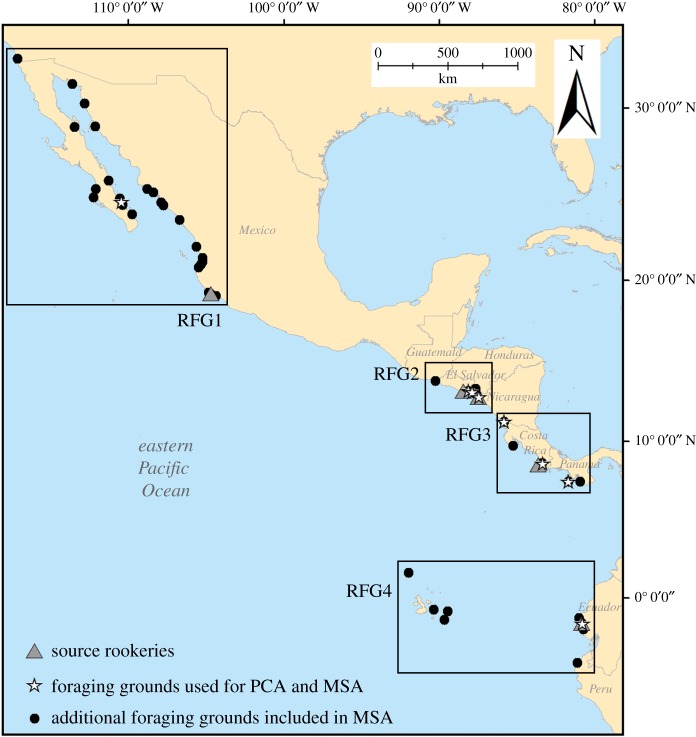
Sample collection sites, including source rookeries (grey triangles), foraging grounds with sample sizes in the range *n* = 20–117 (white stars) that were used in principal components analysis to determine regional foraging grounds (RFGs; black boxes), as well foraging grounds with sample sizes in the range *n* = 1–7 (black circles) that were also included in each RFG.

We collected tissue samples from hawksbill turtles at foraging grounds along the eastern Pacific rim (figure 1) between 2004 and 2015 using a combination of direct monitoring (i.e. tangle nets or manual dive captures), strandings and fisheries by-catch monitoring activities. We used rookery samples from Gaos *et al.* [29], which were supplemented by seven additional rookery samples. When feasible, we measured the curved carapace length (CCL) for all hawksbills encountered and applied Inconel flipper tags (National Band and Tag, Newport, KY, USA) to allow for ongoing identification. After collection, samples were placed in vials containing greater than 95% ethanol or water saturated with sodium chloride, which were subsequently stored in a −20°C freezer.

We amplified a 766 bp segment of mtDNA from the control region of hawksbill tissue samples following the protocol outlined in Gaos *et al.* [29]. Haploytpes (KT934080, KT934070, KT964296, KR012503, KR012504, KT003685, KT072795, KR012505, KT072797, KU695258, KU695259, KX646708, KT934051) were determined using Geneious v. R8 (Biomatters Inc., Newark, NJ, USA) and aligned against sequences in a local reference library, as well as those found in the GenBank database (http://www.ncbi.nlm.nih.gov).

### 2.2. Data analysis

We calculated *F*_ST_ values for rookeries and foraging grounds using Arlequin v. 3.5.1.2 [30]. Because we were interested in identifying broad-scale genetic patterns and had variable sample sizes (mean *n* = 12.4, s.d. = 24.8) from disparate foraging grounds, we pooled samples to create regional foraging grounds (RFGs). To determine which samples would be included in each RFG, we conducted a principal components analysis (PCA) in the package GenAlEx v. 6.5 [31] on *F*_ST_ values for eight foraging grounds for which we had sample sizes in the range *n* = 20–117 (figure 1). The samples from the foraging grounds in each PCA cluster were then combined with neighbouring foraging grounds for which we had sample sizes in the range *n* = 1–7 (figure 1).

We used Bayesian mixed stock analysis (MSA) in the software ‘BAYES’ [32] to estimate the contribution of rookeries to each RFG. The two rookeries in RFG2 were combined into a single regional estimate due to both their proximity and genetic similarity. Average annual number of nests at rookeries in RFG1 = 6.9 (±7.3), RFG2 = 187.6 (±50.7), RFG3 = 52.0 (±n.a.) and RFG4 = 22.3 (±10.0) [33]. For the MSA, we ran a total of four Markov chain Monte Carlo (MCMCs) using uniform priors, coinciding with the number of potential source rookeries, with each MCMC consisting of 50 000 steps initiated at different starting points. We used a burn-in of 25 000 steps and calculated the posterior distribution from the remaining 25 000 for all chains, then ran the Gelman and Rubin shrink factor diagnostic to test that all chains had converged (i.e. less than 1.2) [32]. Individuals with haplotypes not observed in rookeries (i.e. orphan haplotypes, *n* = 5, 0.9% of total samples) were removed from the study.

## 3. Results

**Figure 2.**
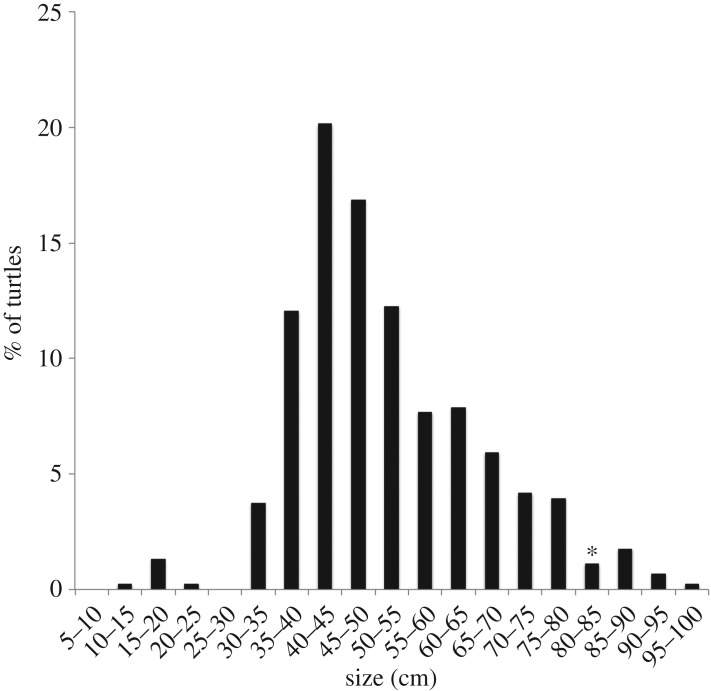
Curved carapace length (CCL) distribution bins (centimetres) of hawksbills analysed in this study. Asterisk indicates bin containing average CCL for nesting female hawksbills in the eastern Pacific [34].

We obtained 766 bp sequences from the mtDNA control region for hawksbill turtles samples collected at 45 foraging grounds (*n* = 535) and five rookeries (*n* = 267) in nine countries (figure 1). Samples collected at foraging grounds included hawksbills with average CCL 51.4 cm (s.d. = 14.1, range 14.0–95.0, figure 2).

Our PCA identified four distinct geographical clusters (see electronic supplementary material, figure 1 for PCA results), leading to the creation of four RFGs (mean sample size *n* = 133.8, s.d. = 78.7). *F*_ST_ values indicated highly significant (*p* < 0.001) structure among all potential source rookeries except for the two located in RFG1 and RFG4. However, these rookeries are separated by greater than 3500 km and it is extremely unlikely that they are demographically homogeneous [35], thus both were treated as independent source rookeries in our MSA.

**Figure 3.**
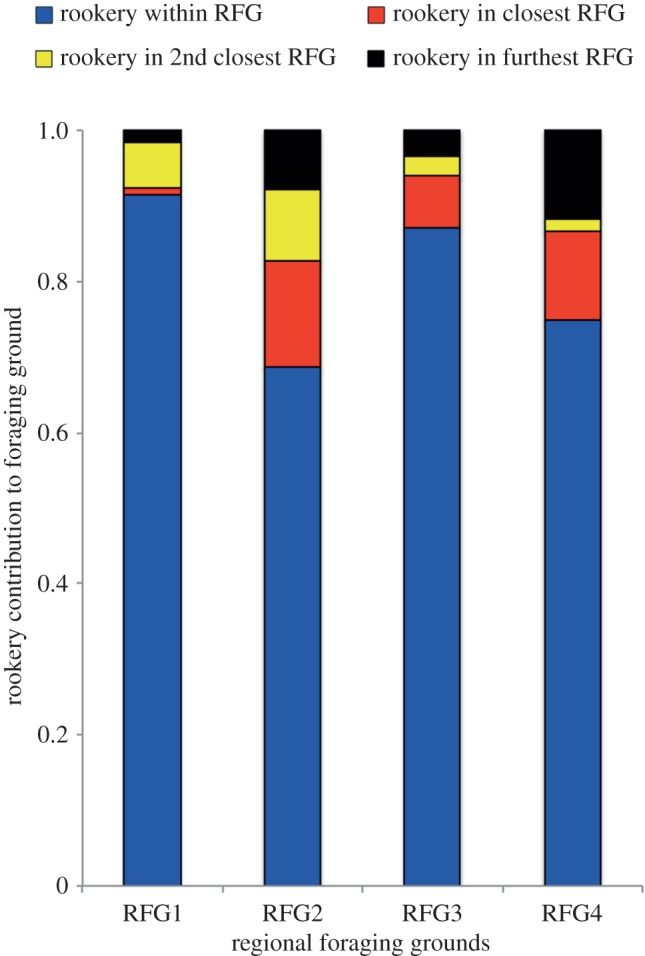
Results of the Bayesian mixed stock analysis showing the contributions of rookeries based on location (represented by bar colour) to the four regional foraging grounds (RFGs). Nearest rookeries (blue bars) contributed the majority of foraging turtles to the RFG in which they were found.

Results of our MSA indicated that rookeries located in each RFG (figure 1) contribute the overwhelming majority (average = 80.5%, s.d. = 10.5) of turtles to foraging grounds in that same RFG (figure 3; see electronic supplementary material, table S1 for detailed MSA results).

## 4. Discussion

Although it has long been established that upon reaching sexual maturity, marine turtles return to the vicinity of their natal beaches to nest or reproduce [36], the potential role of philopatry to foraging areas during juvenile stages has remained largely overlooked as a general life-history trait within the taxon. Our results indicate that juvenile hawksbills in the eastern Pacific use foraging grounds in the region of their source rookeries, a pattern we term natal foraging philopatry (NFP). Despite major differences in the size of our source rookeries, i.e. annual nests among rookeries varied in some cases by an order of magnitude, our results indicate that rookeries in each region, irrespective of their size, were the primary contributors to foraging grounds in the same regions in which they were found. Similarly, while our results indicate rookery contributions are influenced by distance, our findings demonstrate that rookeries in each region contributed virtually all turtles to foraging grounds in those same regions (figure 3). This finding was consistent even for RFGs that were in close proximity to one another (e.g. RFG2 and RFG3).

Our results confirm recent studies that found evidence of NFP in Atlantic juvenile loggerhead, green and hawksbill turtles [16–22]. Combined, these findings suggest that natal philopatry for marine turtles extends beyond mating and nesting adults, and that NFP is a common strategy exhibited across life stages for a number of marine turtle species and stocks. NFP has not been described in all species, e.g. leatherback sea turtles (*Dermochelys coriacea*), yet the pervasiveness of the NFP pattern is particularly notable given that it can manifest even in species and populations known to experience a panmictic post-hatchling dispersal pattern (e.g. [16,20,37]).

Although the NFP pattern is clearly observed in eastern Pacific hawksbills, what is not clear is the role of potential mechanisms behind the NFP pattern in this and other species or stocks, which could include oceanic currents, geomagnetic imprinting, visual and chemical cues, genetic memory, habitat associations, learned migration behaviour and other factors [9,15,38–40]. Our findings of NFP and previous field research [26,27,29,33,41] suggest that eastern Pacific hawksbills may not conform to the commonly postulated open-ocean dispersal theory in which post-hatchling turtles are believed to leave natal areas via entrainment in offshore currents [7,9,37], which for hawksbills would be major ocean currents of the eastern Pacific [42]. Instead, we raise the possibility that hawksbill in the eastern Pacific remain in coastal regions during the post-hatchling phase, categorized by Bolten [9] as a Type 1 hatchling developmental pattern, or undergo a truncated pelagic dispersal phase that keeps them out of major offshore currents. In contrast with marine turtle populations in other parts of the world, hawksbills along Pacific Central America nest at rookeries located within inshore mangrove estuaries [43,44] and these systems are strongly regulated by tidal fluctuations. Coupled with the reduced swim gaits exhibited by hawksbills compared with other marine turtle species [45], these currents could maintain hawksbill hatchlings closer to shore during the initial post-hatching phase in the eastern Pacific [46]. Recent research of lobster gillnet fisheries that operate in neritic waters (i.e. less than 1 km from shore) off Pacific Central America has led to the documentation of various hawksbills with CCL measuring 15 cm or less in size [41], further supporting the Type 1 hatchling development model as a potential mechanism underlying NFP for hawksbills in the eastern Pacific. To date, the flatback turtle (*Natator depressus*) is the only species of marine turtle whose hatchlings have been identified as not having a pelagic phase during early post-hatchling development [9,41,47], a pattern that has been largely attributed to the inshore location of their nesting beaches [46]. At present, data on fine-scale currents are not available for the coastal zones of the eastern Pacific where hawksbills are found, which limits opportunities to conduct meaningful particle modelling efforts. Future research combining particle modelling (e.g. [12,14]), tracking of post-hatchlings (e.g. [34]) and satellite telemetry of young life-stage (6–12 months old) juveniles (e.g. [15,37]) would help elucidate the post-hatchling movements of hawksbills in the eastern Pacific.

Irrespective of the mechanism, affinity to foraging grounds in proximity to rookeries would provide several selective advantages, namely higher lifetime reproductive success from more frequent visits to rookeries [48], as well as higher survival as the absence of large-scale migrations between rookeries and foraging grounds would reduce some threat exposure [4,14,49]. Recent telemetry and nesting biology research of female hawksbills in the eastern Pacific confirms relatively short post-nesting migrations or non-migratory behaviour altogether [26], as well as relatively common annual nesting intervals [29].

Our findings have important implications for life-history research and conservation of marine turtles, particularly for hawksbills in the eastern Pacific. NFP may mean that addressing local threats to foraging hawksbills has potentially greater conservation value for nearby rookeries and hence local populations. Similarly, whereas the conservation of marine turtles can often require the collaboration of governments from multiple nations over large ocean regions [49–51], NFP of hawksbills in the eastern Pacific indicates that one or two neighbouring nations may be able to protect the majority of the life cycle of an entire stock or management unit. Additionally, large rookeries are often recognized for their importance to sustaining marine turtle populations [52] by provisioning a ‘storage effect’, in which via one or two solid recruitment classes per generation they can enable repopulation of an ocean region on ecological timescales [29,53]. However, findings of NFP and the strong stock structure among RFGs indicate that rookeries in each RFG maintain unique genetic contributions, which suggests that focusing conservation efforts solely on larger rookeries would be misguided.

As NFP appears common among many marine turtle species, focused research on the ubiquity of this pattern in individual populations is needed to advance marine turtle ecology and to support effective conservation management strategies. Similarly, NFP as a general life-history concept merits specific discussion and inclusion in current marine turtle and spatial ecology literature. Finally, further research into NFP for other wide-ranging aquatic species known to exhibit nesting philopatry, such as various salmonid and seabird species (e.g. [54,55]), may also be warranted to support robust conservation planning.

## Supplementary Material

Supplemental Figure 1.

## Supplementary Material

Supplemental Table 1

## Data accessibility

The input files used in the MSA are available via the Dryad repository at: (http://dx.doi.org/10.5061/dryad.38t23) [56]. The sequence files used in this analysis (GenBank accession numbers: KT934080, KT934070, KT964296, KR012503, KR012504, KT003685, KT072795, KR012505, KT072797, KU695258, KU695259, KX646708, KT934051) are available through the GenBank database (http://www.ncbi.nlm.nih.gov).
