# Supplementary figures and images for: Natal foraging philopatry in eastern Pacific hawksbill turtles
Source: R Soc Open Sci. 2017 Aug 23;4(8):170153. doi: 10.1098/rsos.170153 (PMC5579084; doi:10.1098/rsos.170153)

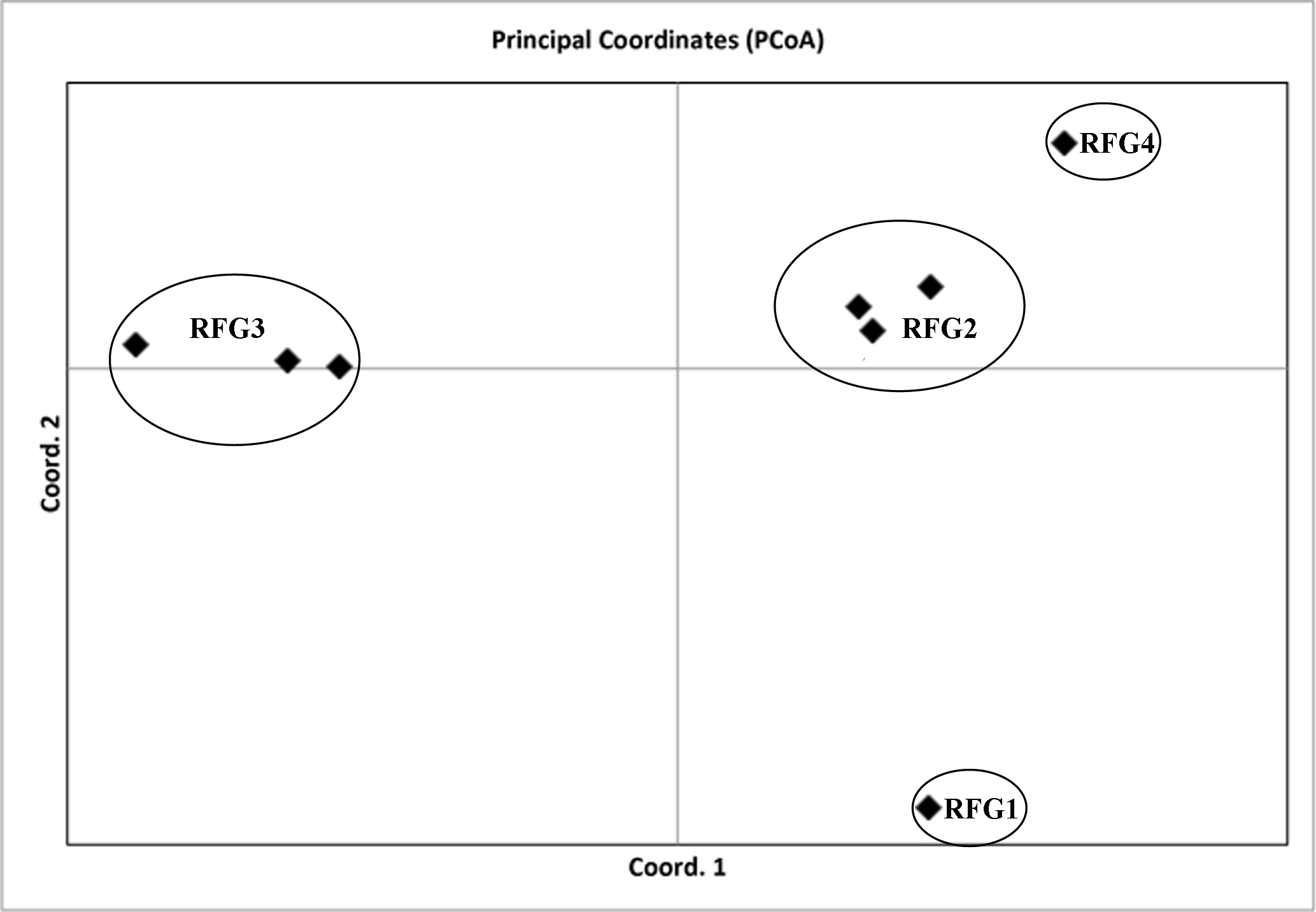

Supplement: Supplemental Figure 1. [file rsos170153supp1.tif]
